# Effect of the Mode of Fermentation on the Behavior of *Penicillium bilaiae* in Conditions of Abiotic Stress

**DOI:** 10.3390/microorganisms11041064

**Published:** 2023-04-19

**Authors:** María Vassileva, Vanessa Martos, Luis F. García del Moral, Nikolay Vassilev

**Affiliations:** Instituto de Biotecnología, Universidad de Granada, 18071 Granada, Spain

**Keywords:** *Penicillium bilaiae*, stress abiotic factors, different modes of fermentation, P-solubilisation activity

## Abstract

The ability of a *Penicillium bilaiae* strain to support acid production and simultaneously solubilize inorganic sources of phosphate in conditions of submerged, solid-state fermentation (SSF) and immobilized cell system was examined in this study. Abiotic stress factors such as NaCl and different values of pH were introduced into the different fermentation process schemes to measure the fungal response. The results showed a higher tolerance of *P. bilaiae* when the fermentation process was carried out in solid-state and immobilized-cell conditions, which mimics the natural state of the soil microorganisms. The acidic culture conditions were not found to be suitable for fungal growth, which increased at a higher pH, with values of 4.0 and 6.0 being optimal for all types of fermentation. The presence of increasing amounts of NaCl provoked low biomass growth, titratable acidity, and simultaneous phosphate (P) solubilization. These results were, however, less pronounced at pH 4.0 and 6.0, particularly in conditions of SSF. Studying stress-tolerant microbial characteristics, particularly in different conditions and combinations of stress factors, is of great importance for further managing the overall microbial inoculants’ production and formulation process as well as their applications in specific soil–plant systems.

## 1. Introduction

Plants are able to reach optimum growth and production in the absence of stress conditions. Low and high temperature, soil salinity, drought, acidity, and heavy metals are considered the most important abiotic environmental factors that affect both plant physiology and growth. Although different strategies have been experimented regarding the protection of plants in conditions of different stresses, the application of soil microbes is accepted as an effective tool for the alleviation of different stresses. In a recent review, the relationship between abiotic factors, the soil microbiome, and plants has been analyzed [1,2]. In general, microorganisms, as one of the key components of both natural and cultivated soils, are shown to affect the soil quality and plant productivity in harsh environments [3], and rebuilding soil productivity by introducing selected plant beneficial microorganisms is expected to increase soil microbial species richness [4]. Particularly important are biofertilizers based on plant growth-promoting bacteria and fungi. A wide range of soil microbes are known to alleviate soil stresses on plant growth and yield production, but the latter group is known for its high tolerance to salinity and pH extremes, and the active release of siderophores, indole acetic acid, ligno-cellulases, chitinase, gibberellins, and organic acids [5,6]. It has been suggested that soil salinity (soluble mineral salts in soils on a volume or weight unit) and sodicity (concentration of Na^+^ ions) can affect nutrient acquisition by plants and soil physical–chemical properties (including pH) [7]. Understanding the complexity of soil–plant–microbe interactions in the rhizosphere, and particularly the role of microorganisms, on soil fertility and crop quality and productivity, and how they tolerate environmental stresses, is now of great importance [8,9]. In this sense, one of the criteria for the selection of commercial formulated microbial products should be stress-tolerance. On the other hand, biotechnological methods should be developed and employed to protect biofertilizing microorganisms, even with stress-tolerant properties and in soil extreme conditions, in order to enhance their effects when more than one stress factor is present [10,11]. Due to climate change and human activities, soil salinity is causing desertification in Mediterranean countries including Spain, Italy, Portugal, Greece, and France [12]. Depending on the soil type, salinity affects the soil pH value. In general, soil acidification negatively affects soil fertility, declining plant growth, reduces nutrient bioavailability, and provokes soil degradation [13]. Particularly in degraded ecosystems, soils characterized by stress abiotic factors, including pH and high salinity, affect the activity of phosphate (P)-solubilizing microorganisms, thus decreasing the level of soluble phosphate [12]. Therefore, it is of great importance to study in vitro the behavior of plant-beneficial microorganisms in conditions of different levels of salinity and pH as individual stress factors or in combination. This approach could be important for selecting stress-tolerant, plant-beneficial microorganisms, and further to determine production schemes and formulation procedures [8]. It has repeatedly been shown that biofertilizers offer several benefits such as improved soil fertility and health, enhanced nutrient availability, plant protection against soil-borne pathogens, enhanced biotic and abiotic stress tolerance, and less environmental pollution compared to chemical fertilizers [14]. Biofertilizers can be produced and formulated by applying different fermentation processes and formulation procedures, respectively [10]. Fermentation processes can be in solid-state and submerged conditions. In the latter case, microorganisms in free state or immobilized on/in different carriers can be employed [11]. In this short report, the effect of NaCl and pH, combined at different values, is tested by applying *Penicillium bilaiae* grown in solid-state and submerged fermentation processes in the presence of animal bone char as a source of inorganic *P. bilaiae* was used in this study as it was one of the first fungal P-solubilizing commercial products with proved efficiency [15]. Animal bone char was used as an alternative to the rock phosphate, the latter being listed amongst the 30 critical raw materials and amongst the five materials with both high supply risk and high economic importance [16]. Gel-entrapped *P. bilaiae* was also tested using Ca-alginate as a carrier as it is the most-used cell-embedding gel carrier.

## 2. Materials and Methods

### 2.1. Microorganism

*Penicillium bilaiae*, identified by morphologic and physiologic characteristics, was used throughout this study. The strain belongs to the microbial collection of the Department of Chemical Engineering, University of Granada. Cultures were maintained on potato dextrose agar (PDA; Merck, Darmstadt, Germany) grown at 28 ± 2 °C in the dark and stored at 4 °C in Petri dishes.

### 2.2. Fermentation Processes

#### 2.2.1. Solid-State Fermentation (SSF)

Experiments in conditions of SSF were performed in 300 mL Erlenmeyer flasks with 10 g of dry sugar beet wastes (particles of 1 mm) moistened by adding 5 mL potato–dextrose broth (PDB) per g dry substrate. The fungal inoculum was prepared by collecting conidia from 6-day-old cultures grown on PDA in a 0.1% (*v*/*v*) Tween 80 solution, and 1 mL of a conidial suspension with 10^6^ conidia/mL was used as inoculum. Incubation of the fungal culture was carried out at 28 °C for 10 d in static conditions.

#### 2.2.2. Submerged Fermentation

The fermentation in conditions of submerged liquid process was carried out in 250 mL Erlenmeyer flasks containing 50 mL fermentation medium (PDB) inoculated with 1 mL (10^6^ spores per mL) spore suspension prepared from 6-day-old PDA-grown culture. This experiment was carried out with orbital shaker ThermoForma (Fisher Scientific, New York, NY, USA).

#### 2.2.3. Immobilized Cell Fermentation

Spores from 6-day-old *P. bilaiae*, suspended in sterile distilled water, were shaken by mixer. Then, 10 mL of the spore suspension with 2 × 10^6^ spores mL^−1^ were mixed in 3% sodium alginate solution and then extruded in 250 mL of 0.5 M calcium chloride solution. Calcium alginate beads of 2 mm were allowed to harden for 30 min under gentle stirring at 28 °C. Then, 30 alginate beads were transferred to 250 mL Erlenmeyer flasks containing 50 mL of PDB medium.

The incubation of freely suspended or immobilized *P. bilaiae* was carried out at 28 °C using an orbital shaker (ThermoForma, 150 rev min^−1^) for 7 d or 3 d, respectively. In the latter case, after the first batch the medium was removed, the immobilized material was washed with sterile distilled water and 100 mL of fresh medium was added to each flask with the alginate-entrapped fungus. Five repeated-batch cycles with the immobilized cells were carried out for 72 h following the same cultivation conditions and procedure.

In all treatments, 3% animal bonechar (ABC, BES Ltd., Birmingham, Scotland) was added. Sodium chloride and different levels of pH were applied in all treatments. The pH was adjusted using 1 N HCl and 1 N NaOH before sterilization.

### 2.3. Analytical Methods

The free fungal biomass grown in submerged and solid-state conditions was collected and determined by weight loss after incineration at 500 °C for 6 h to avoid the weight of phosphate particles. The assessment of gel-entrapped fungal mass in the beads was performed by dissolving 0.5 g of cell-gel beads (30 beads) in 1% (*w*/*v*) sodium citrate solution and magnetically stirring for 30 min. After the dissolution of the beads, the fungal biomass was separated with vacuum filtration with a 3 μm cellulose acetate filter (Millipore, SSWP04700, Darmstadt, Germany) and weighed after drying overnight at 60 °C.

Aqueous extracts were obtained from the 1 g solid-state process sample by the addition of distilled water up to a 10:1 (*v*/*w*) ratio. The extraction was assisted mechanically with an Ultra-Turrax homogenizer (MERCK, Z732346, IKA-Werke, Staufen, Germany) followed by centrifugation.

Samples of the cultivation media were centrifuged (Sorvall S16, Thermo Fisher, New York, NY, USA) at 8000 g for 20 min, and the supernatant was used for the measurements of soluble P, pH, and titratable acidity. Soluble P was determined spectrophotometrically (Shimadzu-1601-PC, Kyoto, Japan) using the vanadate–molybdate reagent (Fluka Cat. No 94685). Titratable acidity was determined by titrating 1.5 mL of supernatant to pH 7 with 0.1 M NaOH using bromothymol blue as the indicator. The pH was measured with Mettler Toledo pH meter S-400 (Basel, Switzerland).

All the experiments were conducted in triplicate and data presented as mean ± SE. One way ANOVA with post hoc Tukey HSD was conducted to analyze statistically significant differences among various treatments.

## 3. Results and Discussion

Biomass growth, titratable acidity and P-solubilization rate by *P. bilaiae* were studied when simultaneously exposed to salt stress at different values of pH and salinity, and at different modes of fermentation. It should be noted, however, that in this study the immobilized fungal system did not support the presence of NaCl. In general, as the bead shape is an important parameter in the survival and functionality of entrapped microorganisms, even partially destroyed beads are strongly associated with a protrusion of the growing cells [17]. Gel-cell beads should be mechanically strong in the fermentation conditions where shared forces and substances such as phosphate, sodium, and potassium could destroy their structure [18]. Therefore, in the case of immobilized *P. bilaiae*, only different values of pH were tested. Low acid production and simultaneous ABC solubilization were detected at pH 2 (data not shown). Increasing the pH in the medium resulted in unfavorable growth conditions in the medium, particularly at the first cycle and in the external part of the alginate cell-beads (microscopic view of bead slices). However, the biomass at pH 4.0 increased during the first three repeated batch cycles, and then was stabilized at 0.475–8 g/g carrier with an increase of 16.3% comparing the first and third–fifth cycle (Table 1). Similar patterns of the growth profile were observed at pH 6.0 and 8.0, but the difference in the biomass growth between the first cycle and the last three cycles was 20% and 27%, respectively. pH values of 4.0 and 6.0 resulted in the highest amount of solubilized phosphate, which dropped after the third batch cycle to reach its lowest value at the last fermentation cycle.

SSF is known for its unique conditions among microorganism, solid particles, liquid, and air, allowing a higher growth of filamentous fungi in comparison with submerged fermentation [19]. In a previously reported study on the conditions of solid-state cultivation, it was concluded that NaCl has a well-demonstrated antimicrobial activity on filamentous fungi, causing slower growth rates and longer lag phase durations compared to the NaCl-free control [20]. Similarly, in this experiment (Table 2) the growth of *P. bilaiae* was the highest at the treatment without NaCl independently of the pH value. The production of titratable acidity by *P. bilaiae* cultured on sugar beet wastes moistened by PDB showed its highest value of 48–49 mmol with the simultaneous solubilization of inorganic P (183–210.5 mg/L) obtained at pH 4.0 and 6.0. The tendency was for both of these activities to decline at low/high pH from being alkaline to acidic (pH 6.0–4.0 to 2.0) and from slightly acidic to alkaline (pH 6.0 to 8.0). The highest concentration of biomass of 59.9–64.1 mg/g solid substrate was registered at pH 4.0 and 6.0 without addition of NaCl.

Likewise, *P. bilaiae* could also tolerate salt concentrations in submerged fermentation experiments, although a decline was observed in the biomass concentration, particularly at higher salt concentration and higher values of the pH (Table 3). At pH 2.0, low acidity and solubilization activity were registered which at pH 4.0 increased. However, a simultaneous drop in the titratable acidity and the P-solubilization rate was also observed when increasing the concentration of NaCl in the medium.

In general, *P. bilaiae* tolerated different values of pH and salinity in all experiments in different fermentation conditions. The most suitable mode of fermentation was the solid-state process, followed by immobilized fungal culture. Other, similar studies have observed that the application of SSF possesses several biotechnological advantages such as higher fermentation productivity, a higher end concentration of products and lower catabolic repression [21]. Zhang et al. [22] suggested that the advantages of SSF were associated with the more rapid rate of cell growth and better fluidity and permeability of the cell membrane. Similarly, immobilized cells are characterized by general advantages such as an ability to synthesize various useful chemicals by specific biochemical reactions, and the possibility of repeated/continuous activity based on prolonged catalytic life [23]. In fermentation and soil conditions, immobilized cells systems avoid the wash-out of cells, ensure higher cell concentration in small volumes, and the slow release of cells [11]. Advantages of immobilized cell formulations for agricultural applications also include an excellent protection of cells from adverse environmental effects. Both these types of fermentation processes simulate the natural state of the soil microorganisms in soil embedded in polysaccharide-based biofilms or attached on/in soil particles [24].

## 4. General Discussion and Conclusions

It is now widely accepted that agricultural production is strongly influenced by climate-change-derived factors such as low/high temperature, pH, and salinity, which are considered to be the most important abiotic environmental stress conditions. The latter also affects plant growth and the metabolic activity of plant-beneficial soil microorganisms. A better understanding of the interrelation between plants and microorganisms within the rhizosphere and their effects on soil fertility, crop quality and productivity as affected by environmental stresses is of great importance. Equally important is to select, formulate, and apply microorganisms with stress-tolerant characteristics, to enhance their overall plant-microbe efficacy and particularly plant adaptation and resistance towards abiotic stress factors. In natural, unstressed conditions, the presence of fungal microorganisms such as *Aspergillus*, *Penicillium*, and *Trichoderma* in the rhizosphere directly/indirectly affects the plants via their morphological and biochemical characteristics. However, under stress conditions plants could reduce their growth; in particular, high salinity provokes considerable morphological, biochemical, and physiological changes [25]. Similarly, it is well known that soil pH affects processes that are interlinked with the geological and chemical aspects of the soil environment [26]. Soil acidity affects nutrient cycling and availability and determines the toxicity of metals [27]. Both salinity and pH determine soil microbial community characteristics [28]. The interaction of plant-beneficial microorganisms with host plants resulted in multiple effects such as enhanced stress tolerance, root modification, enhanced soil quality characteristics, improved nutrient uptake, and the suppression of pathogens. *Penicillium*, *Trichoderma*, and *Aspergillus* spp. are reported to follow the above scheme and significantly increased plant biomass and growth parameters and increased nutrient acquisition [29]. Fungal microorganisms also reduce the sodium toxicity in plants under salinity and drought stress, when compared with control plants [29]. During the last 1–2 decades, the importance of soil enrichment with plant beneficial microorganisms was repeatedly shown, particularly to substitute different chemical products and also to ensure healthy food products. Their effect was higher in poor and disturbed soils where the restoration of the soil fertility was necessary. The main procedures of the development of formulated products with plant beneficial microorganisms include the selection of the target microorganisms, the characterization of the morphological, physiological and biochemical profile of the selected strains, the selection and optimization of the fermentation process, the formulation of the active biomass components, and the development of an application scheme. Therefore, a part of these procedures includes studies on the stress tolerance of the strains. There are a great number of publications on bacteria and fungi, studying their responses in stressed fermentation process conditions. However, rarely do these studies include a combination of stress factors and/or a comparison of different modes of fermentations. For example, in alkaline soils typical for southern Spain, salt content and pH have collinearity, as mentioned by other authors recently [24]. In this work, we also tried to show the effect of the above conditions on the P-solubilization activity of the studied strain. *P. bilaiae* was tolerant to the applied concentration of NaCl, different values of pH, and combinations of them. Maximum P-solubilization and biomass were obtained in the absence of NaCl and decreased when increasing its concentration. The concentration of NaCl up to 3% did not inhibit the solubilization of phosphorus from ABC, although it was less. In general, the P solubilization activity was associated with the capacity of the tested microorganisms to acidify the culture medium, which is normally explained by the production and release of low molecular weight organic acids (titratable acidity) and protons excretion [30]. There was a decrease in solubilization activity with an increase in sodium chloride concentration. This might be either due to lower biomass fungal growth or the neutralization of the acidifying agents (protons and acid ions) by chloride ions in the medium. The higher analyzed parameters in both cases, using immobilized cell-based and SSF, could be preferable for the development of formulation/application schemes, but further testing of *P. bilaiae* in soil–plant systems is needed to prove the P solubilization activity of the different forms of the fungal inoculant resulting from different fermentation process profiles in conditions of abiotic stress.

## Figures and Tables

**Table 1:** Effect of pH on biomass growth, titratable acidity, and P-solubilization activity of *P. bilaiae* immobilized in alginate beads.

Batch	pH Initial/Final	Dry Biomass (g/g carrier)	Titratable Acidity (mmol)	P _sol_ (mg/L)
1	4.0/3.78 ± 0.04	0.411 ± 0.07	12.13 ± 0.30	126 ± 4.0
1	6.0/3.63 ± 0.1	0.343 ± 0.04	10.14 ± 0.23	114.9 ± 3.7
1	8.0/3.69 ± 0.1	0.306 ± 0.02	9.44 ± 0.32	103.6 ± 4.1
2	4.0/3.59 ± 0.14	0.458 ± 0.02	12.94 ± 0.30	128.3 ± 7.0
2	6.0/3.43 ± 0.1	0.432 ± 0.04	11.43 ± 0.38	121.9 ± 3.7
2	8.0/3.61 ± 0.1	0.352 ± 0.01	10.54 ± 0.18	110.9 ± 5.2
3	4.0/3.49 ± 0.20	0.478 ± 0.12	13.90 ± 0.34	130 ± 2.8
3	6.0/3.39 ± 0.18	0.430 ± 0.08	12.44 ± 0.29	128.1 ± 5.7
3	8.0/3.42 ± 0.10	0.400 ± 0.02	12.14 ± 0.42	120.6 ± 4.1
4	4.0/3.58 ± 0.04	0.475 ± 0.04	10.53 ± 0.80	114.0 ± 1.0
4	6.0/3.75 ± 0.15	0.425 ± 0.07	10.28 ± 0.30	111.0 ± 1.9
4	8.0/3.79 ± 0.1	0.411 ± 0.02	10.14 ± 0.22	92.6 ± 2.1
5	4.0/3.78 ± 0.04	0.476 ± 0.03	10.20 ± 0.60	91.3 ± 1.0
5	6.0/3.95 ± 0.15	0.429 ± 0.05	8.88 ± 0.20	83.0 ± 1.3
5	8.0/4.11 ± 0.1	0.406 ± 0.06	8.14 ± 0.23	79.6 ± 1.2

**Table 2:** Effect of pH and salinity on biomass growth, titratable acidity, and P-solubilization activity of *P. bilaiae* in conditions of solid-state fermentation. Mean ± standard error (*n* = 3) is presented.

pH_init_	NaCl (%)	Biomass * (mg/g)	Titratable Acidity (mmol)	pH Final	P_sol_ (mg/L)
	0	44 ± 1.1 a	22.90 ± 0.26 a	2.69 ± 0.01	134.0 ± 3.1 a
	0.75	34 ± 1.3 ab	14.09 ± 0.15 b	2.97 ± 0.03	119.2 ± 9.3 b
2	1.5	32 ± 1.3 b	12.17 ± 0.26 b	2.92 ± 0.02	115.7 ± 6.8 b
	2.25	30 ± 0.9 bc	10.61 ± 0.23 c	3.00 ± 0.02	101.5 ± 0.7 c
	3	28 ± 1.0 c	9.91 ± 2.42 c	3.17 ± 0.08	88.8 ± 7.5 d
	0	59.9 ± 2.0 a	48.94 ± 4.58 a	2.81 ± 0.09	183.7 ± 10.0 a
	0.75	50.4 ± 1.3 ab	41.40 ± 0.46 b	2.97 ± 0.01	152.4 ± 7.5 b
4	1.5	48.3 ± 1.1 b	40.53 ± 0.53 b	2.99 ± 0.02	146.9 ± 6.5 b
	2.25	43.1 ± 0.7 c	38.47 ± 1.73 c	3.01 ± 0.02	136.0 ± 8.6 c
	3	39.9 ± 0.4 c	35.20 ± 1.40 c	3.12 ± 0.02	121.9 ± 4.5 d
	0	64.1 ± 3.0 a	48.64 ± 1.85 a	2.86 ± 0.08	210.5 ± 13.7 a
	0.75	60.9 ± 1.8 b	47.00 ± 4.25 a	2.92 ± 0.06	197.6 ± 2.8 b
6	1.5	55.7 ± 2.3 b	51.31 ± 0.70 a	3.05 ± 0.0	171.3 ± 7.5 c
	2.25	47.0 ± 2.1 c	51.31 ± 3.21 a	3.12 ± 0.02	155.0 ± 12.7 d
	3	44.1 ± 1.3 c	47.65 ± 1.61 a	3.09 ± 0.03	136.3 ± 4.6 e
	0	42 ± 0.6 a	13.34 ± 0.46 a	2.92 ± 0.03	124.95 ± 10.79 a
	0.75	31 ± 1.1 b	11.95 ±0.3 ab	3.10 ± 0.03	112.16 ± 5.68 b
8	1.5	29 ± 0.3 bc	10.59 ± 0.26 b	3.05 ± 0.01	103.58 ± 4.84 c
	2.25	26 ± 1.0 bc	10.75 ± 0.91 b	3.15 ± 0.01	95.08 ± 11.92 c
	3	24 ± 0.5 c	9.55 ± 0.95 c	3.17 ± 0.01	88.64 ± 11.62 bcd

* Biomass is mg dry biomass per g of initial dry substrate. All data were subjected to a one-way analysis of variance (ANOVA). Values not sharing a letter are significantly different at *p* < 0.05 (Tukey’s test).

**Table 3:** Effect of pH and salinity on biomass growth, titratable acidity, and P-solubilizing activity of *P. bilaiae* in conditions of submerged fermentation. Mean ± standard error (*n* = 3) is presented.

pH_init_	NaCl (%)	Dry Biomass (g/flask)	pH_final_	Titratable Acidity (mmol)	P_sol_ (mg/L)
2	0	0.259 ± 0.003 a	2.48 ± 0.01	5.4 ± 0.09 a	14.7 ± 2.4 a
	0.75	0.227 ± 0.003 a	2.59 ± 0.01	1.8 ± 0.15 b	4.7 ± 0.9 b
	1.5	0.202 ± 0.011 a	2.54 ± 0.02	0.58 ± 0.23 c	2.2 ± 0.8 c
	2.25	0.200 ± 0.010 a	2.62 ± 0.01	0.43 ± 0.09 c	2.7 ± 0.8 c
	3	0.196 ± 0.012 b	2.66 ± 0.02	0.0 ± 0.01 d	1.1 ± 0.7 cd
4	0	0.397 ± 0.009 a	3.18 ± 0.04	11.13 ± 0.3 a	106 ± 4.0 a
	0.75	0.338 ± 0.005 a	3.48 ± 0.05	7.68 ± 0.09 b	62.8 ± 0.9 b
	1.5	0.320 ± 0.021 a	3.50 ± 0.01	7.70 ± 0.23 b	55.1 ± 1.3 c
	2.25	0.327 ± 0.014 a	3.50 ± 0.02	7.83 ± 0.23 b	56.3 ± 0.7 c
	3	0.307 ± 0.004 b	3.57 ± 0.04	6.90 ± 0.09 c	52.9 ± 0.5 c
6	0	0.470 ± 0.005 a	3.49 ± 0.01	10.14 ± 0.23 a	84.9 ± 3.7 a
	0.75	0.406 ± 0.015 a	4.06 ± 0.02	7.69 ± 0.44 b	47.3 ± 1.9 b
	1.5	0.399 ± 0.010 a	3.95 ± 0.03	6.40 ± 0.09 bc	42.6 ± 0.5 bc
	2.25	0.330 ± 0.022 b	3.94 ± 0.01	6.20 ± 0.09 bc	41.4 ± 0.5 bc
	3	0.318 ± 0.037 bc	3.92 ± 0.02	5.89 ± 0.35 c	40.8 ± 0.7 c
8	0	0.446 ± 0.009 a	3.37 ± 0.03	10.28 ± 0.32 a	71.2 ± 5.8 a
	0.75	0.385 ± 0.002 a	3.66 ± 0.01	7.83 ± 0.32 b	44.4 ± 0.7 b
	1.5	0.377 ± 0.003 a	3.65 ± 0.01	6.83 ± 0.18 b	42.2 ± 0.4 b
	2.25	0.333 ± 0.010 ab	3.70 ± 0.01	5.17 ± 0.15 bc	38.8 ± 1.0 b
	3	0.321 ± 0.018 abc	3.71 ± 0.01	4.74 ± 0.15 c	38.1 ± 0.6 b

All data were subjected to a one-way analysis of variance (ANOVA). Values not sharing a letter are significantly different at *p* < 0.05 (Tukey’s test).

## Data Availability

Not applicable.
